# Articulated Non-Rigid Point Set Registration for Human Pose Estimation from 3D Sensors

**DOI:** 10.3390/s150715218

**Published:** 2015-06-29

**Authors:** Song Ge, Guoliang Fan

**Affiliations:** School of Electrical and Computer Engineering, Oklahoma State University, Stillwater, OK 74078, USA; E-Mail: song.ge@okstate.edu

**Keywords:** point set registration, visible points extraction, segment volume validation, human pose estimation

## Abstract

We propose a generative framework for 3D human pose estimation that is able to operate on both individual point sets and sequential depth data. We formulate human pose estimation as a point set registration problem, where we propose three new approaches to address several major technical challenges in this research. First, we integrate two registration techniques that have a complementary nature to cope with non-rigid and articulated deformations of the human body under a variety of poses. This unique combination allows us to handle point sets of complex body motion and large pose variation without any initial conditions, as required by most existing approaches. Second, we introduce an efficient pose tracking strategy to deal with sequential depth data, where the major challenge is the incomplete data due to self-occlusions and view changes. We introduce a visible point extraction method to initialize a new template for the current frame from the previous frame, which effectively reduces the ambiguity and uncertainty during registration. Third, to support robust and stable pose tracking, we develop a segment volume validation technique to detect tracking failures and to re-initialize pose registration if needed. The experimental results on both benchmark 3D laser scan and depth datasets demonstrate the effectiveness of the proposed framework when compared with state-of-the-art algorithms.

## 1. Introduction

Human pose estimation is an important research topic in the field of computer vision and pattern recognition, which has been actively studied for decades [1]. In recent years, with the rapid development of various 3D sensing technologies, such as laser scanners and the affordable RGB-D depth sensors (e.g., Kinect from Microsoft), human pose estimation is attracting more and more attention lately [2,3] due to its wide applications (e.g., digital entertainment [4] and medical diagnostics [5–8]). Although significant progress has been obtained by recent endeavors, human pose estimation from 3D point sets or depth data remains a challenging problem due to several factors, such as the high degree-of-freedom (DoF) of pose parameters, large pose variation, complex motion patterns, body shape variability and imperfect sensor data (noise, outliers, incomplete date caused by self-occlusions and view changes).

Traditional pose estimation approaches are based on 2D images or image sequences captured from one or multiple cameras [9], where 2D image data have inherent ambiguity and uncertainty [10]. Recent research activities are more focused on the point sets or depth maps captured by 3D sensors, which are becoming more affordable and prevalent. These approaches can be roughly divided into three categories, discriminative, generative and hybrid. Discriminative approaches usually involve a learning process, which requires a labeled training dataset to deal with complex shape, pose variability and various motion patterns [11–14]. A large and diverse training dataset is imperative for this kind of approach. Generative ones treat pose estimation as an alignment problem where the objective is to fit a pre-defined template to a target point set. Furthermore, many approaches formulate pose estimation as a point set registration problem where an articulated structure is involved, often with the local rigidity assumption [15–18]. These approaches usually require good correspondence initialization or similar poses between the template and target to avoid being trapped in local minima or use some data-driven features to reduce the search space. Particularly, for sequential pose tracking, previous pose estimation is often used to predict the new pose and/or to initialize the registration in the present frame [18–21]. Hybrid approaches attempt to take advantages of two kinds of approaches by involving a pre-labeled database to provide good pose or correspondence initialization for template-based pose estimation [19,22].

In this paper, we propose a new generative framework for human pose estimation from the perspective of probabilistic point set registration. Our approach is suitable for both 3D point sets (from laser scanners) and sequential depth data (from depth sensors), where there are three main challenges. Correspondingly, we have three main technical contributions in this work. First, it is difficult for the template-based registration to deal with large pose variation in the 3D point sets, which exhibit both articulated and non-rigid deformations globally and locally. We propose a hybrid registration approach to cope with this problem by integrating our recently proposed topology-aware non-rigid registration algorithm, called global-local topology preservation (GLTP) in [23], with a segment-aware articulated iterative closest point (SAICP) adapted from articulated iterative closest point (AICP) [16] to better interface with GLTP results. Specifically, GLTP provides reliable correspondence estimation and segmental labeling that naturally fits with SAICP-based articulated pose estimation. Second, the depth data are often noisy and incomplete due to the self-occlusion and view-changing problems, which fundamentally challenge the registration process. We invoke an efficient visible point extraction scheme to refine and adapt the template sequentially, which improves both the efficiency and accuracy of pose tracking. Third, sequential pose tracking could inevitably have failed frames, which must be detected and corrected to avoid error propagation. We develop a simple, yet effective segment volume validation technique to ensure the robustness and stableness of pose tracking over a long depth sequence. A couple of metrics are defined to validate each segment from the GLTP's output, and the necessary template update or re-initialization is triggered before SAICP is applied. The proposed framework is evaluated both on 3D laser scan data and standard depth data by comparing against several recent algorithms. Our algorithm can achieve state-of-the-art performance in terms of the joint position error at moderate computational complexity.

The rest of this paper is organized as follows. In Section 2, we provide a brief review of the related work in the fields of point set registration and human pose estimation, as well as our research motivation. In Section 3, we present the proposed framework for pose estimation and tracking, where five major steps are discussed in detail along with a complete pseudocode. Experimental results are reported in Section 4, where our algorithm is evaluated on two benchmark datasets and compared against several state-of-the-art algorithms. We draw conclusion in Section 5.

## 2. Related Work

**Figure 1:**
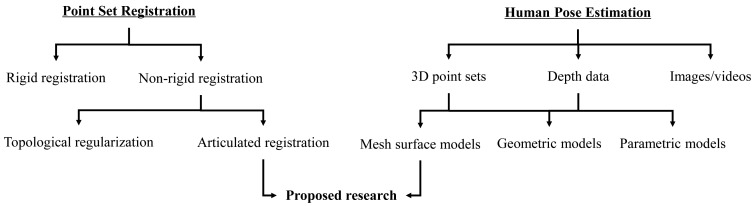
Related work in terms of point set registration and human pose estimation.

We provide a brief overview of the background of this research, which involves two separate, but related topics: point set registration and human pose estimation, as shown in Figure 1. Particularly, we focus on the recent research on human pose estimation from depth data, which has many practical applications due to the recent development of RGB-D cameras and other affordable range sensors.

Point set registration is a fundamental topic for many computer vision tasks. The registration techniques usually fall into two categories: rigid and non-rigid depending on the underlying transformation model. Iterative closest point (ICP) [24,25] is a classic rigid registration method, which iteratively assigns correspondence and then finds the least squares transformation by using the estimated correspondence. For non-rigid registration, shape features are commonly used for correspondence initialization [26–28] or directly involved in the matching process [29,30]. Recently, topology-aware approaches are becoming an important category where a Gaussian mixture model (GMM)-based probabilistic registration strategy is commonly used [23,31–36]. For example, a Gaussian radial basis functions (GRBF)-based displacement function with a global topological constraint, coherent point drift (CPD), was introduced in [33,34], which leads to a powerful and general GMM-based non-rigid registration algorithm. Two kinds of graph-based regularizations, which aim to improve the robustness to outliers and to preserve the intrinsic geometry, were incorporated in the CPD framework [37,38]. In [23], by jointly considering the global and local topological constraints, global-local topology preservation (GLTP) was proposed to deal with non-rigid and highly articulated deformations. As a special case of non-rigid registration, articulated structure registration is an active and interesting research topic due to its wide applications. Most existing approaches assume that the articulated structure is locally rigid (e.g., [16,17]) and often require good correspondence initialization or similar poses between the template and the target to avoid being trapped into local minima [16,22].

On the other hand, traditional human pose estimation research is mainly based on 2D images or videos [9], and there is a dramatic increase of research efforts on pose estimation from 3D data, including point sets and depth maps, due to the availability of various affordable 3D sensors. A key element in the problem of pose estimation is human body representation, and the often used models include mesh surface, geometric and parametric models. In this paper, we focus on the mesh model based representation, and human pose estimation is cast as a point set registration problem. The main challenge here is the large pose and shape variations between the template and observed target models, especially when there is no temporal information available, such as individual 3D laser scan data. An often used remedy to this problem is to involve some training data, an efficient classifier or data-driven features to initialize the registration process. For example, a 3D database, which contains a large number of mesh models along with embedded skeletons, was used in [22] to search for the most similar pose for a given input depth image based on which, CPD is performed for pose estimation by refining correspondences. In [13], the input depth image is matched to the template model by employing a pre-trained regression forest; then, joint positions are estimated by minimizing an energy function from predicted correspondences over the full body. In [39], an upper-body segmentation is first obtained from depth images, which is used to initialize AICP-based pose estimation. Additionally, human pose tracking was also recently studied intensively, which takes advantage of the smooth motion assumption and uses pose estimation in the previous frame to initialize the present one [18–20]. However, sequential depth data usually are noisy and incomplete due to significant self-occlusions and dramatic view changes, which lead to inaccurate or unstable pose estimation. Therefore, some constraints are introduced to improve the reliability of pose estimation. For example, some pose hypotheses are predicted to guide pose estimation in a new frame [19,40], which are created from detected feature points corresponding to anatomical landmarks. In [18,41], the pose hypothesis in the current frame is predicted by a linear third order autoregression model, which involves three previous estimated poses. It is worth mentioning that failure detection is a very important step for pose tracking. Some kinematics and physical constraints are used in [22,42] to detect failures after pose estimation and to make necessary corrections if needed.

Our research is deeply motivated and inspired by the aforementioned endeavors. We are specifically focused on three issues related to some previous limitations. The first is to deal with complex articulated non-rigid deformations caused by large pose and shape variations by a unique hybrid registration approach that does not require correspondence initialization and can deal with large pose variation. The second is to cope with self-occlusions and view changes in pose tracking by invoking a sequential template update strategy that does not require any feature detection or data segmentation. The third is to detect pose tracking failures during (not after) pose estimation by using a new segment volume validation technique after correspondence estimation, which is amenable to represent kinematic and psychical constraints.

## 3. Proposed Framework

**Figure 2:**
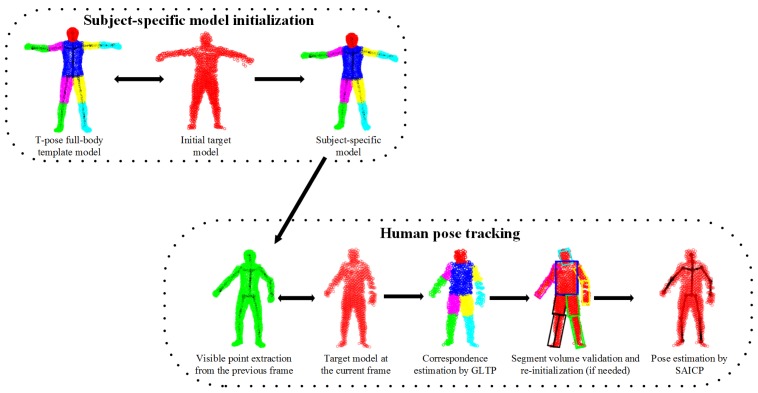
Overview of the proposed human pose tracking framework.

An overview of the proposed framework is shown in Figure 2, which involves five steps. First, we learn a subject-specific articulated model to initialize the body shape and size for a new subject. Second, visible point extraction is performed from the subject-specific model to create a partial template model, which either involves previous pose estimation or a “T-pose” template. Third, our recently proposed non-rigid registration algorithm (GLTP) is used for correspondence estimation from the observed target model. Fourth, segment volume validation is invoked to detect tracking failures and to trigger pose re-initialization if needed. Last, segment-aware AICP (SAICP) is used for articulated pose estimation by refining correspondence estimation at each segment iteratively. For 3D point sets, only Steps 1, 3 and 5 are needed; while for depth sequences, sequential pose tracking will involve all steps, and Steps 1, 2, 3 and 5 will support frame-by-frame pose estimation.

### 3.1. Subject-Specific Shape Initialization

A personalized articulated shape model is important for accurate and robust pose estimation due to the large body shape and size variabilities between the template and a target model. In [20], the personalized body shape represented by vertices of a given mesh is jointly controlled by a low-dimensional shape parameter vector learned from a laser scan database and a pose parameter vector through linear blend skinning. These shape parameters are obtained by optimizing a local cost function, which considers both Euclidean and the norm-based distances between matched points. In [18], after a global scaling initialization, the template shape is adapted sequentially after frame-wise pose estimation by segment-level size estimation and shape refinement along the norm direction.

In this work, we learn a subject-specific articulated model in two steps by involving a standard “T-pose” template **Y** (*M* × *D*) that represents *M D*-dimensional points {**y***_m_*|*m* = 1, …,*M*} and an initial target **Z** (*N* × *D*), which denotes *N D*-dimensional points {**z***_n_*|*n* = 1, …, *N*} from a subject (with four limbs fully stretched under a normal standing pose). Both **Y** and **Z** are preferred to have similar poses. Specifically, **Y** is extracted from a human mesh model with pre-labeled body segments and an articulated skeleton. **Z** is captured by a 3D sensor that should reflect a naturally stretched pose where most joints are revealed for accurate shape initialization.

In the first step, we apply the coherent point drift (CPD) algorithm [34] for non-rigid registration between **Y** and **Z**. CPD is a powerful Gaussian mixture model (GMM)-based registration approach, which enforces the GMM centroids to move coherently as a group to preserve the topological structure of the point set. The core of the CPD algorithm is that it defines the non-rigid transformation as a displacement function in a reproducing kernel Hilbert space (RKHS) with the spatial smoothness regularization defined as the Fourier domain norm. Additionally, it also proved that the optimal displacement function is represented by a linear combination of Gaussian kernel functions as:

(1) T(Y,W)=Y+GW

where **G***_M_*_×_*_M_* is the Gaussian kernel matrix with element $g_{ij}=\exp(-\frac{1}{2}{\left\|\frac{{\text{y}}_{i}-{\text{y}}_{j}}{\beta }\right\|}^{2})$, *β* is the kernel width and **W***_M_*_×_*_D_* is the weight matrix. The regularization term of **W**, which encourages global coherent motion, is defined as:

(2) $$E_{\text{CPD}}{(\text{W})=\text{Tr}(\text{W}}^{T}\text{GW})$$

where Tr(**B**) denotes the trace of the matrix **B**. The solution of **W** can be achieved by an iterative expectation maximization (EM) algorithm. Since **Y** and **Z** do not have large pose variation, CPD could provide reliable registration results along correspondence estimation between the two point sets.

**Figure 3:**
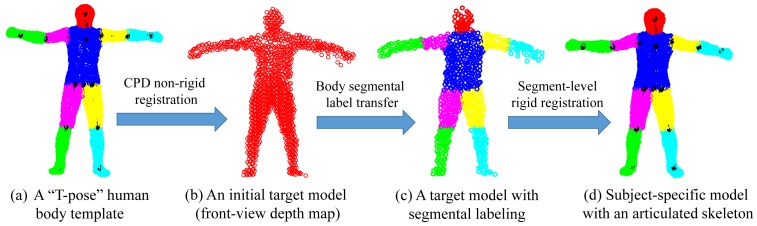
Subject-specific articulated body shape initialization.

In the second step, we bake a skeleton in **Z** by transforming the skeleton of **Y** via segment-level rigid registration according to the estimated correspondences. As a result, a subject-specific articulated shape model **Ẑ** is learned that plays an important role for future pose estimation. In the case of depth data with incomplete front-view point sets, we introduce visible point extraction (to be discussed in the following) to obtain a front-view template prior to CPD registration. Then, after segment-level rigid registration, invisible parts will be transformed along with their visible counter parts to build a complete subject-specific model **Ẑ**. An example of subject-specific shape initialization is shown in Figure 3.

### 3.2. Visible Point Extraction

**Figure 4:**
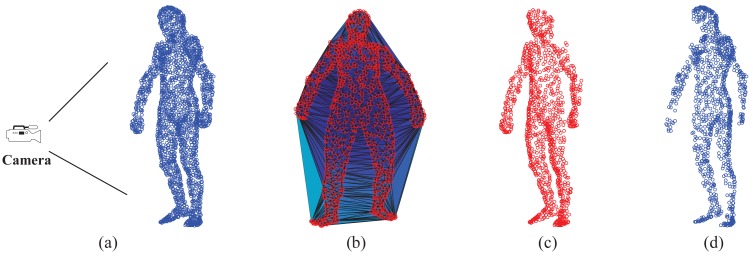
(**a**) Relative position between the camera and the 3D template; (**b**) The inverted points lie in the convex hull; (**c**) The extracted visible points; (**d**) The invisible points.

Visible point extraction is important to support depth map-based pose estimation, especially in the case of sequential depth data. This step requires the relative position between the full-body template model and the camera. In this work, we use the hidden point removal (HPR) operator [43] to detect visible points of a given template model. Given a point set **A** = {**a***_i_*} and the viewpoint *C* (camera position), the HPR operator mainly has two steps to determine ∀**a***_i_* ∈ **A** whether **a***_i_* is visible from *C*. In the first step, we associate with **A** a coordinate system and set *C* as the origin. Then, we find the inverted point of each a*_i_* using spherical flipping [44] with the following equation:

(3) $${\hat{\text{a}}}_{i}={\text{a}}_{i}+2(R-\left\|{\text{a}}_{i}\right\|)\frac{{\text{a}}_{i}}{\left\|{\text{a}}_{i}\right\|}$$

where *R* is the radius of a sphere, which is constrained to include all **a***_i_*. We denote the set of inverted points by **Â** = {**â***_i_*}. In the second step, we construct the convex hull of **S** = **Â** ∪{*C*}. Then, we can mark a point **a***_i_*, which is visible from *C* if its inverted point **â***_i_* lies in **S**. An example of visible point extraction is shown in Figure 4. After this process, we can obtain the visible point set $\hat{\text{Z}}={\left\{{\hat{\text{z}}}_{m}\right\}}_{m=1}^{M_{vis}}$ of the full-body template model that is ready to perform the registration.

### 3.3. Topology-Aware Non-Rigid Registration

The objective of this step is to estimate correspondences between a labeled template point set and any target point set with an arbitrary pose. This is critical for latter SAICP-based articulated pose estimation. Because the subject-specific model **Ẑ** may not be in a strict fully-stretched “T-pose”, it may not serve as a good template here. Therefore, in the case of individual point set registration, we always use the standard “T-pose” template, where all body segments are fully stretched, as shown in Figure 3a. In the case of sequential depth data, we can either use the standard “T-pose” template for every frame by treating each frame independently or invoke a tracking strategy by creating a new template from the pose estimation result of the previous frame. The latter one is more computationally efficient, but must be accompanied with tracking failure detection and may require re-initialization if needed. As those used in Section 3.1, we still use **Y** and **X** to denote the template and a new target point set, respectively, in the following.

Due to the possible highly articulated non-rigid deformation in **X**, traditional registration algorithms (e.g., CPD) may not be able to provide reliable correspondence estimation. Therefore, in this work, we use our previously proposed GLTP algorithm [23], which unifies two topologically complementary constraints, *i.e.*, CPD-based global motion coherence and local linear embedding (LLE)-based local topology [45], into a GMM-based probabilistic registration framework. Specifically, the CPD-based motion coherence defined in Equation (2) is helpful to keep the overall spatial connectivity of a multi-part point set during the registration process, and the LLE-based local topological constraint is useful to preserve the neighborhood structure during non-rigid deformation. In this work, we present GLTP in the context of human pose estimation. For each point in **Y**, the local neighborhood is represented by the weighted linear combination of its pre-selected *K* nearest neighbors where the weights are obtained by minimizing the reconstruction error. Then, the LLE-based regularization term has the form:

(4) $$E_{\text{LLE}}(\text{W})={\overset{M}{\underset{m=1}{\text{∑}}}\left\|\left({\text{y}}_{m}+\text{G}(m,\cdot )\text{W}\right)-\overset{K}{\underset{i=}{\text{∑}}}{\text{L}}_{mi}\left({\text{y}}_{i}+\text{G}(i,\cdot )\text{W}\right)\right\|}^{2}$$

where **G** is the Gaussian Kernel with coefficients matrix **W** shown in Equation (2), which controls the transformation, **G**(*m*, ·) denotes the m-th row of **G** and **L** is the weight matrix containing the neighborhood information for each point in **Y**. The optimal **W** to preserve the local neighborhood structure is obtained by minimizing Equation (4). Following the general GMM formulation [46] and incorporating two regularization terms, the objective function of GLTP can be written as:

(5) $$\begin{array}{ll} Q(\text{W},\sigma^{2}) & =\overset{M,N}{\underset{m,n=1}{\text{∑}}}p^{\text{old}}(m|{\text{x}}_{n}\frac{{\left\|{\text{x}}_{n}-[{\text{Y}}_{m}+\text{G}(m,\cdot )\text{W}]\right\|}^{2}}{2\sigma^{2}}+\frac{N_{p}D}{2}\ln(\sigma^{2})\\ & +\frac{\alpha }{2}E_{\text{CPD}}(\text{W})+\frac{\lambda }{2}E_{\text{LLE}}(\text{W}) \end{array}$$

where *σ*^2^ is the isotropic variance of all Gaussian components, *α* and λ are two trade-off parameters controlling the GMM matching term and topological constraint terms, *D* = 3 in this work and $N_{P}=\Sigma_{n=1}^{N}\Sigma_{m=1}^{M}p^{\text{old}}(m|{\text{x}}_{n})$ and *p^old^*(*m*|**x***_n_*) are the posterior probabilities from previousGMM parameters:

(6) $$p^{\text{old}}(m|{\text{x}}_{n})=\frac{\exp(-\frac{1}{2}{\left\|\frac{{\text{x}}_{n}-({\text{y}}_{m}+\text{G}(m,.)\text{W})}{\sigma^{\text{old}}}\right\|}^{2})}{{\text{∑}}_{i=1}^{M}\exp(-\frac{1}{2}{\left\|\frac{{\text{x}}_{n}-({\text{y}}_{i}+\text{G}(i,\cdot )\text{W})}{\sigma^{\text{old}}}\right\|}^{2})+c}$$

where *ω* (0 ≤ *ω* ≤ 1) is the weight of a uniform distribution to account for outliers and $c=\frac{{\left(2\pi \sigma^{2}\right)}^{\frac{D}{2}}\omega M}{(1-\omega )N}$. We rewrite the objective Equation (5) in matrix form, take the derivative of it with respect to **W** and set it equal to zero; then, **W** can be obtained by solving a linear system:

(7) $$[d(\text{P}1)\text{G}+\sigma^{2}\alpha \text{I}+\sigma^{2}\lambda \text{MG}]\text{W}=\text{PX}-(d(\text{P}1)+\sigma^{2}\lambda \text{M})\text{Y}$$

where **I** denotes the (*M*×*M*) identity matrix, **P** (*M* × *N*) records the probability of correspondences between template **Y** and target **X** and **M** = (**I** — **L̂**)(**I** — **L̂**)*^T^* where **L̂** is an expansion matrix of L by filling zeros to reshape into a square matrix (*M*×*M*). As detailed in [23], the solution of **W** and *σ*^2^ of GLTP can be obtained by an iterative EM algorithm extended from the one used for CPD optimization. Matrix **P** will used to initialize SAICP-based (segment-aware AICP) articulated pose estimation, to be discussed latter.

### 3.4. Segment Volume Validation

Although sequential pose tracking is efficient in dealing with depth sequences, it is important to validate the tracking result at every frame to prevent the error from propagating over frames. This step is especially important when there are significant and frequent self-occlusions due to dramatic pose and view changes. Traditionally, tracking validation is done based on the pose estimation results by applying some kinematic or physical constraints [22,42]. We propose an effective approach to detect tracking failures at an earlier stage (after GLTP and before SAICP). We first obtain the labeled point set **X̂** from a given input point set **X** by transferring segment labels according to estimated correspondences. We then validate the segment volume for each body segment in **X̂** represented by the minimum volume oriented bounding box (OBB) [47,48], where two metrics are involved as follows.

**Figure 5:**
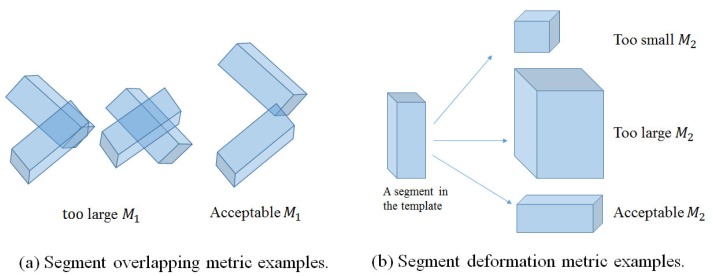
Illustration of two metrics used for segment volume validation.

Segment overlapping metric (*M*_1_): This metric checks the overlapping ratios between every two body segments represented by OBBs in a labeled point set **X̂** of *P* segments, as defined below:

(8) $$M_{1}({\text{S}}_{i})=\underset{j\neq i}{\max}\frac{V(B({\text{S}}_{i})\cap B({\text{S}}_{j}))}{V(B({\text{S}}_{i}))}$$

where **S***_i_* and **S***_j_* (*i, j* = 1, …, *P*) denote two body segments in **X̂**, *B*(**S***_i_*) represents the OBB of **S***_i_* and *V*(·) is the volume of an OBB (*i.e.*, the total number of points). We compute *M*_1_ (**S***_i_*, **S***_j_*) by calculating the percentage of the points, which belong to both **S***_i_* and **S***_j_*, over the total number of points in **S***_i_*. A large value of *M*_1_(**S***_i_*) implies a significant overlap between **S***_i_* and other segments, indicating inaccurate correspondence estimation (Figure 5a).

Segment volume deformation metric (*M*_2_): This metric measures the volume deformation of a segment after GLTP-based non-rigid registration:

(9) $$M_{2}({\text{S}}_{i})=\frac{V(B({\text{S}}_{i}))}{V(B({\text{S}}_{i}^{*}))}$$

where **S***_i_* and ${\text{S}}_{i}^{*}$ are the same body segment in the target **X̂** and in the template **Ẑ**, respectively. As shown in Figure 5b, a small value of *M*_2_(**S***_i_*) indicates that **S***_i_* in **X̂** has missing parts, while a large value of *M*_2_(**S***_i_*) implies that **S***_i_* mistakenly includes some points from other segments. Specifically, because the torso has a relatively stable 3D volume during pose tracking, we use the torso height to replace the volume in Equation (9) in order to enhance the sensitivity of the torso's *M*_2_.

**Figure 6:**
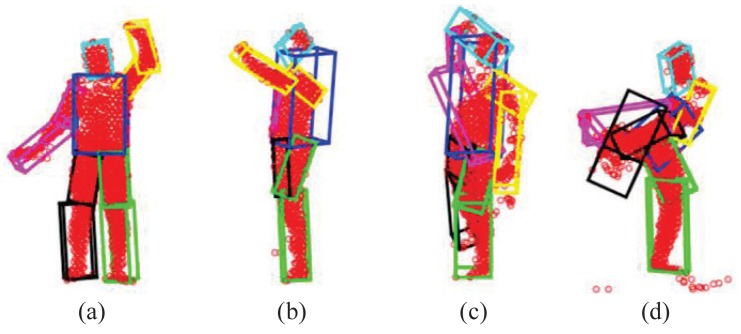
Some examples of segment volume validation: (**a**) a passing case; (**b**) Case I failure (invalid *M*_1_); (**c**) Case II failure (invalid *M*_1_ and *M*_2_) in a couple of limbs and the torso; (**d**) Case III failure (invalid *M*_1_ and *M*_2_ in most segments).

As shown in [23,49], GLTP works very well in most depth sequences we tested, but there are still three possible challenging cases for which GLTP may fail with invalid correspondence estimation, as shown in Figure 6: (1) Case I: some segments become invisible in the current frame due to the view change (e.g., the subject is making a turn from the frontal view to the side view, Figure 6b); (2) Case II: some segments suddenly reappear after being absent for some frames due to the view change (e.g., the subject is turning to the frontal view from the side-view, Figure 6c); (3) Case III: there are significant self-occlusions between two adjacent frames due to large pose variation and fast motion, which causes a large number of missing points in the target point set (e.g., the subject is making a quick high kick, Figure 6d). We will discuss how to detect these three cases by the two proposed metrics and how to remedy accordingly. The thresholds of *M*_1_ and *M*_2_ are given in the experiment.

- The first case can be detected if *M*_1_ is too large for a particular segment or the number of points in a segment becomes too small (e.g., less than 25%). Correspondingly, we update the template obtained from the previous frame by declaring this segment “invisible” and then re-perform GLTP-based non-rigid registration. As shown in Figure 6b, there are significant overlaps between the right arm (purple) and the torso (blue) and between the right (black) and left (green) legs. To mitigate this problem, those segments will not be involved during GLTP registration for re-initialization, and they will deform along with their parents according to their rotations in the previous frame during the latter articulated registration.
- The second case can be checked by both using *M*_1_ and *M*_2_. When there are a couple of limbs that were occluded in previous frames and re-appear in the current frame, those limbs will be likely overlapped with other segments, leading to large *M*_1_ for those reappearing segments. Furthermore, part of the reappearing segments could be mistakenly included in wrong segments (the torso in most cases) whose volumes become much larger, leading to large *M*_2_. As shown in Figure 6c, the reappearing right arm (purple) is merged into the torso (blue), resulting in large *M*_1_, and meanwhile, both the torso and head (cyan) have a large volume change to cover part of the right arm. The remedy for this case is to use the “T-pose” template to re-perform GLTP-based registration for re-initialization.
- The third case is the “worst case scenario” when most segments have invalid *M*_1_ and *M*_2_. This case is very rare in practice, and it is usually due to large self-occlusions, as shown in Figure 6d where the right upper-leg (black), the right arm (purple) and part of the torso (blue) are occluded when the subject is making a quick high kick. In this case, registration-based approaches usually will not work well, and we invoke a simple, yet effective approach to recover the underlying pose by imposing pose continuity across frames and by introducing physical constraints in the step of articulated registration to be introduced in the next section.

### 3.5. Articulated Registration for Pose Estimation

This last step involves two labeled point sets. One is the labeled target **X̂** of an arbitrary pose, and the other is the subject-specific model **Ẑ**, which is expected to have the same body shape and size as **X̂**. The goal is to perform pose estimation of **X̂** by matching with **Ẑ**, which includes *P* rigid body segments {**S**_1_, ⋯, **S**_*P*_} connected by the skeleton model. Because both **X̂** and **Ẑ** are registered with the “T-pose” template **Y**, we can initialize their correspondences by referring to the same template. Then pose estimation is converted to find the rigid transformation for each body segment **S***_p_* (*p* = 1, …, *P*), which can be represented collectively by:

(10) $${\text{T}}_{p}^{W}={\text{T}}_{\text{root}}^{W}\cdots {\text{T}}_{\vee (p)}^{L}{\text{T}}_{p}^{L}$$

where ∨(*p*) denotes the index of the parent of **S***_p_*, ${\text{T}}_{\text{root}}^{W}$ is the transformation of the root in the world coordinate and ${\text{T}}_{p}^{L}$ is the local transformation of segment **S***_j_* with respect to its joint connecting with the parent. ${\text{T}}_{p}^{W}$ could be obtained by minimizing the objective function as:

(11) $$Q\left({\text{T}}_{1}^{W},\cdots \;,{\text{T}}_{P}^{W}\right)=\overset{P}{\underset{p=1}{\text{∑}}}\overset{M_{p}}{\underset{m=1}{\text{∑}}}{\left\|{\text{T}}_{P}^{W}{\hat{\text{z}}}_{m}^{p}-{\hat{\text{x}}}_{m}^{p}\right\|}^{2}$$

where *M_p_* is the number of points in **S***_p_* and ${\hat{\text{x}}}_{m}^{P}\in \hat{\text{X}}$ is the correspondence of ${\hat{\text{z}}}_{m}^{p}\in {\text{S}}_{p}$. A direct optimization of Equation (11) is difficult due to its non-linearity and high-dimensional pose parameters.

**Figure 7:**
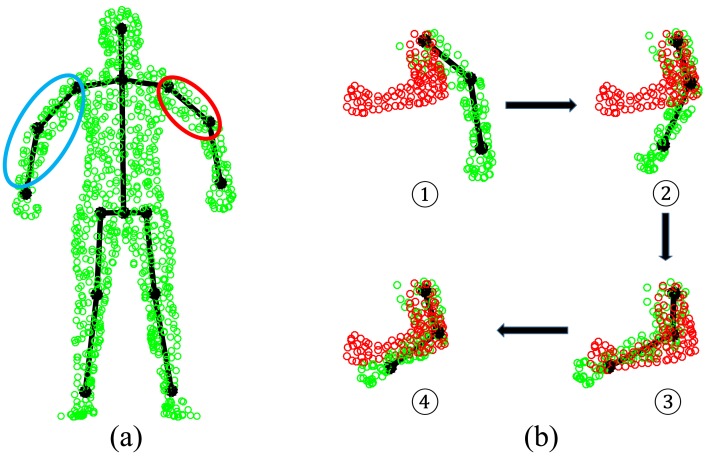
The illustration of the proposed segment-aware AICP (SAICP)-based registration algorithm. (**a**) Two examples to construct the rigid body part: selecting a single segment (red area) or several connected segments (blue area), which cannot be supported by the original AICP algorithm; (**b**) One example of transformation estimation of the left arm. (1) The template (green) and target (red) models; (2) The result of upper-arm deformation; (3) The result of lower-arm deformation; (4) The result of whole-arm deformation.

The original AICP algorithm in [16] adopts a divide-and-conquer strategy to iteratively estimate an articulated structure by assuming that it is partially rigid. In each iteration, the articulated structure is split into two parts by a joint, which is selected randomly or cyclically; then, the classic rigid ICP is performed locally on one of these two parts. AICP works effectively when the template and target have similar segmental configurations (*i.e.*, similar poses), which may not be true in human pose estimation. In our case, given reliable correspondence estimation by GLTP, we follow a more flexible and efficient scheme to construct a partial rigid body part by selecting single or several connected segments. We develop a new segment-aware AICP (SAICP) algorithm to find the rigid transformations for all segments by optimizing Equation (11) in a way that reflects segment-level articulated motion. The main idea is to take advantage of GLTP's output by starting from the root (the torso) and head, which are relatively stable, and then following along the tree-structured skeleton according to the connectivity between segments, as shown in Figure 7a. This allows us to treat the limbs in a particular order, upper, lower and whole, as shown in Figure 7b, and it is efficient to update the rigid transformations of four limbs simultaneously. It is worth mentioning that the correspondences at each segment will be updated during each iteration when the segment label information of **X̂** and **Ẑ** is also used for the minimum distance search.

The SAICP algorithm is discussed in detail as follows. Let Ψ = {**S**_1_, ⋯, **S***_p_*}, which represents a body part composed of *p* (*p* ≤ *P*) connected segments (with *M*_Ψ_, points) along the articulated structure from the labeled target **Ẑ**. We have the objective function defined for this body part as:

(12) $$Q({\text{T}}_{\Psi }^{W})=\overset{M_{\Psi }}{\underset{m=1}{\text{∑}}}{\left\|{\text{T}}_{\Psi }^{W}{\hat{\text{z}}}_{m}^{\Psi }-{\hat{\text{x}}}_{m}^{\Psi }\right\|}^{2}$$

where ${\hat{\text{z}}}_{m}^{\Psi }$ is a point in part Ψ in **Ẑ** and ${\hat{\text{x}}}_{m}^{\Psi }$ is its correspondence in **X̂** that is initialized by GLTP. Classic ICP iteratively updates the correspondence ${\hat{\text{x}}}_{m}^{\Psi }$, and the part-level rigid transformation ${\text{T}}_{\Psi }^{W}$ can be solved in a closed form by minimizing Equation (12). For sequential depth data, visible points are extracted from the template **Ẑ**, which are involved in SAICP to estimate segment-level rigid transformations. In order to preserve the full-body template **Ẑ** during pose tracking, we transform the invisible points of each segment along with their corresponding visible points, so that we always use a pose-specific full-body template at each frame, which is used to initialize a partial template for the next frame estimation via visible point extraction. To ensure a smooth and reasonable tracking result, we impose two constraints for sequential pose estimation. The first is the temporal continuity to ensure that each body segment has a smooth motion trajectory across frames. The second is the physical constraint to avoid the overlapping problem between any two segments. These two constraints are especially useful in the case of large self-occlusions caused by fast motion or significant view changes (e.g., Case III in segment volume validation). The pseudo-code of the proposed pose estimation framework is shown in Algorithm 1.


**Algorithm 1** The Pseudo-Code of the Proposed Pose Estimation Framework.
** Input:** “T-pose” template **Y**, an initial target **Z** and *T* sequential depth frames **X**_1:_*_t_*** Output**: A sequence of deformed full-body models **Ẑ***_t_* (*t* = 1, …, *T*) with estimated joint positions** GLTP Initialization:**
*ω* = 0.1, *K* = 10, *α*_0_ = 10, *β* = 2, λ_0_ = 5 × 10^6^
$${\text{G}}_{ij}=\exp\frac{-1}{2}{\left\|\frac{{\text{y}}_{i}-{\text{y}}_{j}}{\beta }\right\|}^{2}\text{and}\;\text{L}\to \hat{\text{L}}\to \text{M}$$** Shape Initialization:**
  Step 1: CPD-based non-rigid registration between **Y** and **Z**  Step 2: Learn the subject-specific model **Ẑ** by segment-level rigid registration (**Y** ↔ **Z**)  Step 3: Initialize the template for GLTP (non-rigid registration) in the first frame **Y**_0_ = **Y**  Step 4: Initialize the template for SAICP (articulated registration) in the first frame **Ẑ**_0_ = **Z**** For** each depth frame **X***_t_* (*t* = 1, …, *T*) do
  Represent **X***_t_* by a point set $\left\{{\text{x}}_{n}^{t}|i=1,\ldots ,N\right\}$  Visible point extraction to create ${\text{Y}}_{t-1}^{*}$ from **Y**_*t*−1_ (with tracking) or **Y** (without tracking)  
$$\sigma^{2}=\frac{1}{\text{DMN}}{\text{∑}}_{m,n=1}^{M,N}{\left\|{\text{x}}_{n}^{t}-{\text{y}}_{m}\right\|}^{2}$$  GLTP re-initialization for pose tracking: update **G** and **M** according to ${\text{Y}}_{t-1}^{*}$  Correspondence estimation by GLTP between **X***_t_* and ${\text{Y}}_{t-1}^{*}$  While (dissatisfy stopping criteria)E-step:     Compute matrix **P** according to Equation (6).M-step:     Compute weight matrix **W** and *σ*^2^ by solving Equation (7)     (a detailed solution can be found in [23])  End while  According to **P**, **X̂***_t_* that is the labeled **X***_t_* with correspondences can be obtained.    Segment volume validation of **X̂***_t_* and re-initialization if needed according to Equations (8) and (9)  Pose estimation by performing SAICP between **X̂***_t_* and ${\hat{\text{Z}}}_{t-1}^{*}=\left\{{\text{S}}_{p}|p=1,\ldots ,P\right\}$    **For** (*p* from the root to all child segments)     Local ICP for Ψ = {**S***_p_*} by minimizing Equation (13)    **End for**    **While** (stopping criteria not satisfied)     **For** (each of four limbs)      Local ICP for Ψ = {**S***_i_* (the upper-limb only)}      Local ICP for Ψ = {**S***_j_* (the lower-limb only)}      Local ICP for Ψ = {**S***_i_*, **S***_j_*}     **End for**    **End while**  The deformed subject-specific model **Ẑ***_t_* is obtained along with estimated joint positions  In the case of pose tracking, update the GLTP template **Y***_t_* = **Ẑ***_t_* **End for**


## 4. Experiments

Our proposed framework does not involve any training data and is evaluated on two publicly available datasets, 3D SCAPE (Shape Completion and Animation of People) data [11] (captured by a 3D laser scanner) and SMMC-10 (Stanford Time-of-Flight Motion Capture) data [50] (captured by a Swissranger SR4000 time-of-flight (ToF) camera at 25 fps and a resolution of 176 × 144). Below, we present the results corresponding to two datasets, separately.

### 4.1. Experiments for the SCAPE Dataset

#### 4.1.1. Point Set Data Preparation

**Figure 8:**
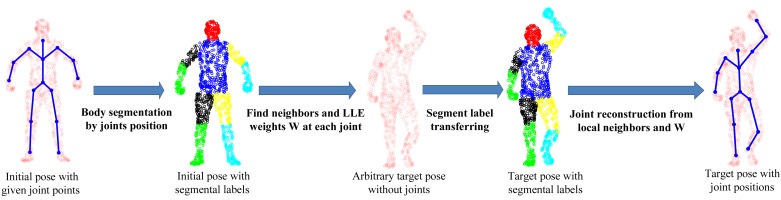
Ground-truth generation of joint positions for SCAPE data.

The SCAPE dataset contains a series of 3D scan data captured from one male subject (the only one publicly available under different poses), which are fully registered (the index of each point stays the same across all poses). It has one initial pose with ground-truth joint positions. To perform quantitative comparative analysis, we develop a simple, yet effective four-step approach to generate the ground-truth joint positions for all other poses, as shown in Figure 8. First, we perform body segmentation for the initial pose according to joint positions. Second, for each joint, we find a set of neighboring points around the joint area between two connected body segments and compute LLE weight coefficients to represent each joint locally. Third, we transfer the segmental labels from the standard pose for any new target pose. Fourth, we use LLE weight coefficients and the associated neighboring points, which share the same indexes as those in the initial pose, to reconstruct each joint position in the target pose. In this way, all poses will have the ground-truth joint positions created for performance evaluation.

#### 4.1.2. Experimental Results: Shape Initialization

**Figure 9:**
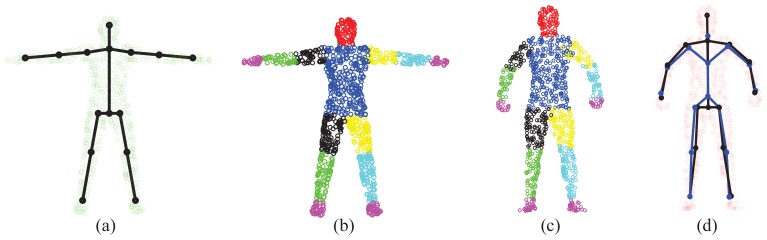
(**a**) The “T-pose” template model used for the SCAPE dataset; (**b**) The labeled template; (**c**) The labeled initial pose; (**d**) The learned subject-specific articulated model for SCAPE data (the estimated skeleton in black and the ground-truth one in blue).

The “T-pose” template used for the SCAPE data is modified from the MotionBuilder humanoid model, which has a skeleton and labeled body segments, as shown in Figure 9a, b, respectively. Given an initial pose from the SCAPE data that is close to the “T-pose”, we use the two-step approach discussed in Section 3.1 for shape initialization. Then, we obtain labeled body segments in Figure 9c and the estimated skeleton (joint positions) in Figure 9d. Compared with the ground-truth skeleton, the average error of joint positions is 2.88 cm. The subject-specific shape model shown in Figure 9d will be used in the following two experiments regarding correspondence estimation and pose estimation.

#### 4.1.3. Experimental Results: Correspondence Estimation

**Figure 10:**
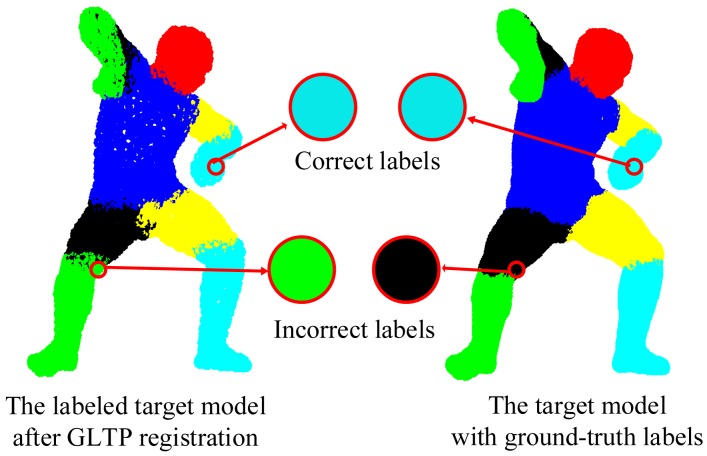
Illustration of the computation of segment labeling accuracy.

We validate the proposed framework on 38 target poses from the SCAPE dataset, most of which have strong non-rigid articulation compared with the template, which makes it a challenging test set. In this case, visible point extraction and segment volume validation are not involved. Since the template and target models are captured from different subjects and also have different numbers of points, it is difficult to obtain the ground-truth correspondences. Thus, a quantitative result in terms of registration error is not available in this experiment. Instead, we use the accuracy of body segment labeling to evaluate the registration performance. During data preparation, we have obtained the ground-truth segment labels for all target poses. For each point in the template model, we propagate its segment label to the corresponding point in the target model by the estimated correspondence. If this assigned segment label is the same as the ground-truth label, we treat it as the correct segment label, as shown in Figure 10. Then, the labeling accuracy for each target pose is calculated as the percentage of the points with correct segment labels over all labeled points.

**Figure 11:**
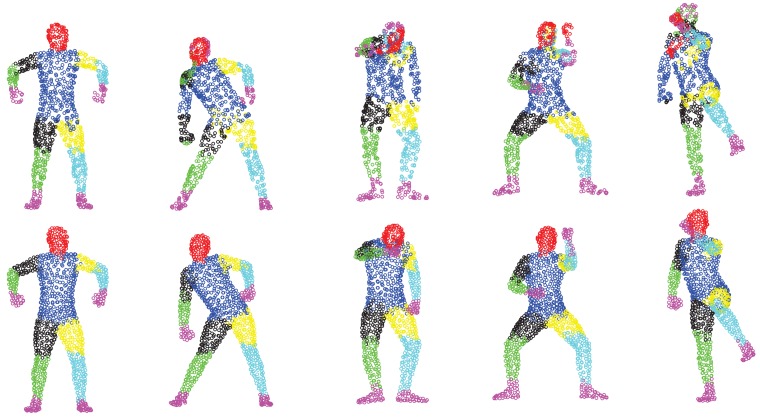
Correspondence estimation: coherent point drift (CPD) results (first row) and global-local topology preservation (GLTP) results (second row).

**Figure 12:**
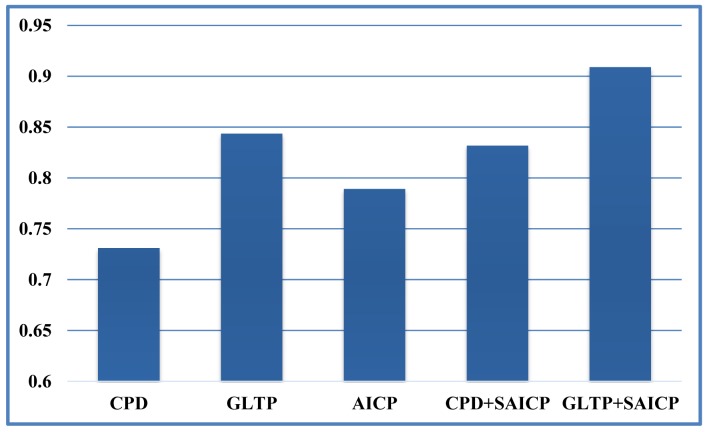
Result comparison on SCAPE data with the labeling accuracy of body segments.

**Figure 13:**
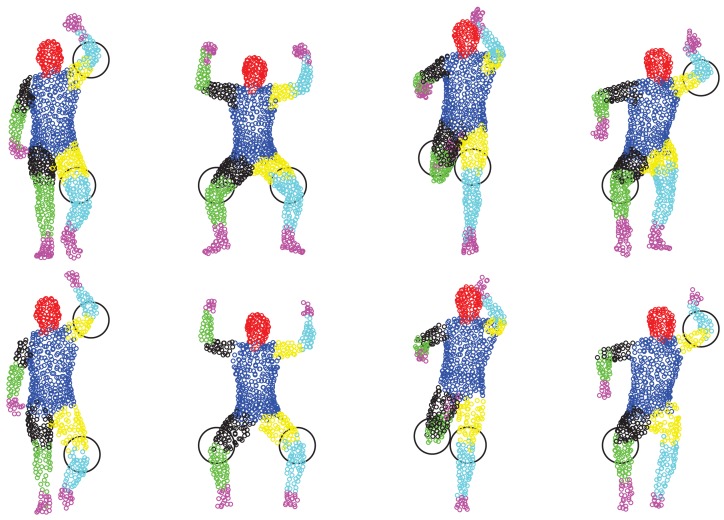
Results of correspondence refinement before (above) and after (below) SAICP, especially around limb joints (circled area).

We first show some qualitative results of GLTP (*α* = 10, *β* = 2, λ = 5 × 10^6^ and *K* = 10) by comparing with CPD in Figure 11 in terms of segment labeling accuracy. When articulated deformation is not significant between the template and target, such as the first pose, both CPD and GLTP perform well. However, in the cases of highly articulated deformations, e.g., Poses 2 to 5, significant labeling errors are observed around the head, limbs and body joints in the CPD results. On the other hand, GLTP provides stable segment label estimation across all poses. However, the results around limb joints are still not very reliable. We further perform the comparative analysis (averaged over 38 poses) with CPD, GLTP and AICP [16] in Figure 12, which shows that GLTP is the best one among all three, and AICP is better than CPD due to the fact that its locally rigid assumption is suitable for 3D human data. Figure 12 shows the labeling accuracy of body segments of our approach (GLTP + SAICP). It is shown that a significant improvement is achieved by using GLTP and SAICP jointly (GLTP + SAICP), which is also better than the one using CPD and SAICP together (CPD + SAICP). We visualize some labeling refinement results in Figure 13, where obvious improvements are seen around limb joints.

#### 4.1.4. Experimental Results: Pose Estimation

**Figure 14:**
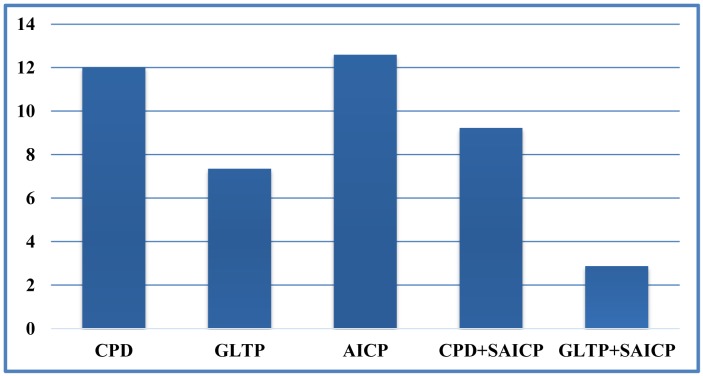
Result comparison on SCAPE data with average joint position errors (cm).

**Figure 15:**
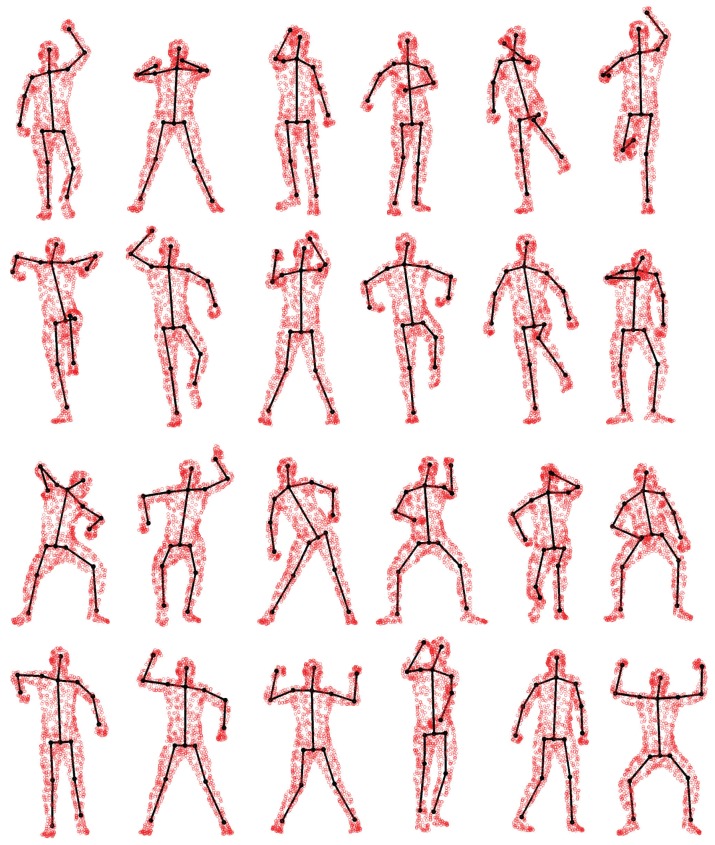
Pose estimation results for some SCAPE data.

We compare pose estimation results in terms of joint position error (cm) in Figure 14. We can see that directly using the estimated corresponding points to compute joint positions cannot achieve a reasonable pose estimation result. Although compared with CPD, GLTP provides much better results, the correspondence estimation around the connection area between two adjacent segments is not reliable due to the lack of segmental information during the registration, which leads to inaccurate pose estimation. As we mentioned before, without a good initialization, AICP is usually trapped into local minima, which results in large estimation errors. Our framework significantly outperforms other options, including CPD, GLTP, AICP and CPD + SAICP, showing the effectiveness of GLTP for correspondence estimation and the necessity of SAICP for pose estimation, which involves the segmental information to refine the GLTP results. We also present some pose estimation results in Figure 15.

#### 4.1.5. Experimental Results: Further Discussion

**Figure 16:**
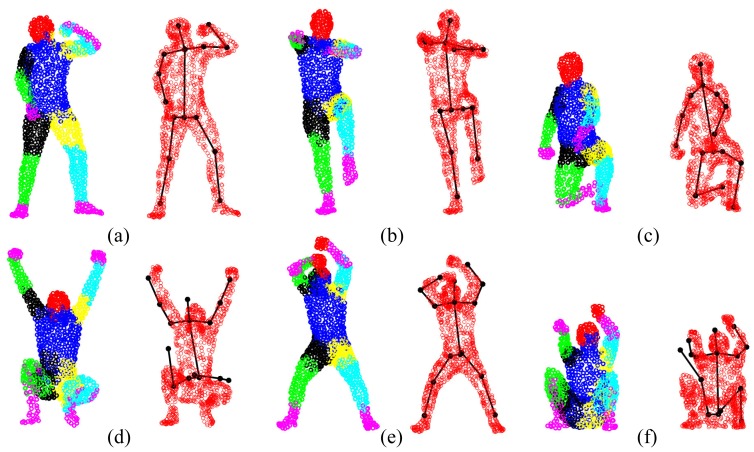
Some challenging cases in the SCAPE data (left: body segment labeling by GLTP; right: pose estimation by SAICP). The left arm/hand (**a,b**) and the right foot/leg (**c**) are mislabeled, which can be corrected during pose estimation. The two legs and feet (**d,f**) and the two hands and head (**e**) are labeled wrongly, which can be partially corrected by pose estimation.

The GLTP registration algorithm, which initializes the correspondences for SAICP-based articulated pose estimation, plays a critical role in the whole flow. Since GLTP uses the Euclidean distance to assign correspondences, it may not be reliable or valid in two challenging cases. First, when there is a strong pose articulation in the point set compared with the standard “T-pose” template, the EM-based GLTP optimization could be trapped into local minima, resulting in some body segments being wrongly labeled, which might be corrected by SAICP during pose estimation. Second, when some body segments are too close (the head and hands) or even merged (lower/upper legs), the shortest distance is no longer valid in those segments, leading to wrong correspondence estimation, which can only be partially corrected by SAICP due to large labeling errors. We further show six challenging cases in Figure 16, where the first row shows three examples of the first case and the second row presents three examples of the second case.

### 4.2. Experiments for the SMMC-10 Dataset

#### 4.2.1. Data Preparation

**Figure 17:**
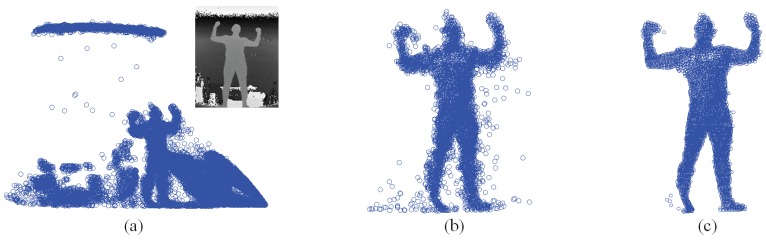
The illustration for depth data pre-processing. (**a**) The point set transferred from a depth image; (**b**) The point set after background subtraction; (**c**) The point set after denoising.

**Figure 18:**
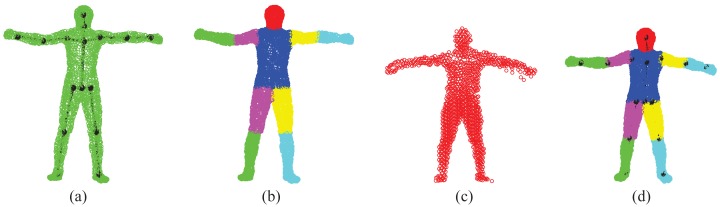
(**a**) The “T-pose” template model for the SMMC-10 dataset; (**b**) The labeled template; (**c**) The initial pose from a depth image; (**d**) The subject-specific articulated model obtained by transforming the template model.

The SMMC-10 dataset contains 28 depth image sequences (numbered 0 to 27) from the same subject with different motion activities, and it provides the ground-truth marker locations. The input depth image cannot be used directly, due to noise/outliers and undesirable background objects. Therefore, we performed three pre-processing steps to make the depth data ready for pose estimation, which include body subtraction by depth thresholding, a modified locally optimal projection (LOP) algorithm for denoising [22] and outlier removal by limiting the maximum allowable distance between two nearest points. Figure 17 shows an example of depth pre-processing for the SMMC-10 dataset. The “T-pose” template (around 2000 points) in this experiment is from [22], which has a built-in skeleton (Figure 18a) along with labeled body segments (Figure 18b). We selected one depth image with “T-pose” from Sequence 6 for shape initialization, which is given in Figure 18c, and the learned subject-specific shape model with a baked-in skeleton and labeled segments is shown in Figure 18d.

#### 4.2.2. Experimental Results: Segment Volume Validation

**Figure 19:**
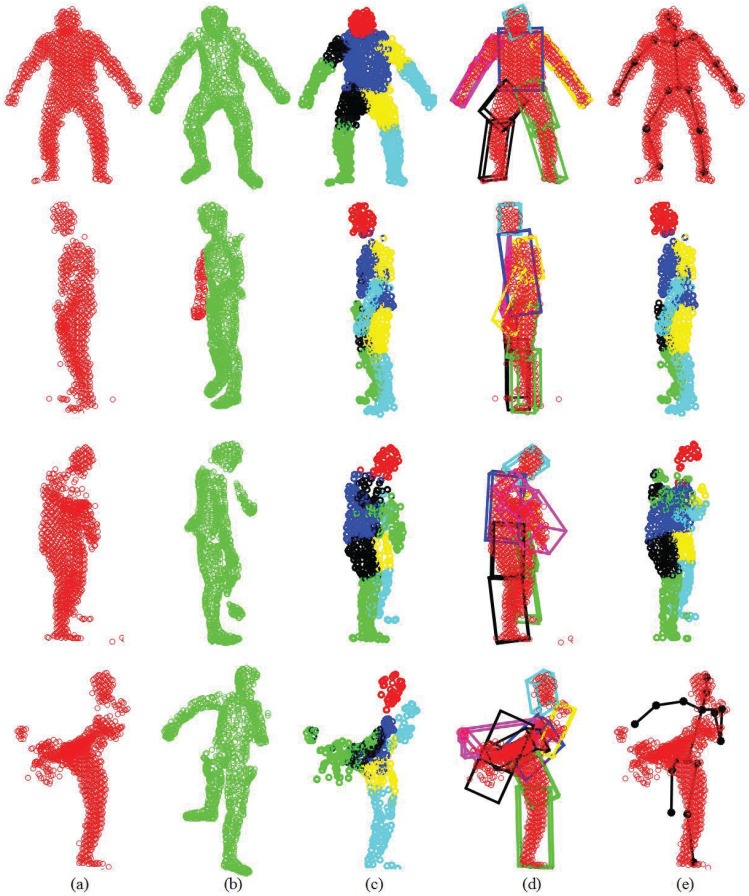
Validation and re-initialization results for a passing case (first row) and three failed cases (second to fourth row). Columns (**a–e**) are the point set in the current frame, that in the previous frame, correspondence estimation results by GLTP (with body segment labels), segment volume validation and pose estimation/re-initialization results, respectively.

In practice, we found that both *M*_1_ and *M*_2_ have very distinct values in the passing and failing cases, indicating their sensitivity for volume validation. In this work, we chose *M*_1_ and *M*_2_ to be 0.3 and 10, respectively. The threshold of the torso's *M*_2_ is 1.4 to reflect the maximum allowable height change. In all 28 testing sequences, the total frame-wise pass rate is over 98%, and there are 1.89% of frames that require re-initialization (Case I or II). Twenty one out of 28 sequences have a 100% passing rate, and Case III is only detected for a few frames in Sequence 25. Some validation examples are given in Figure 19, which shows a passed case (the first row) and three failed cases: (1) In the second row (Case I), the right arm is visible in the previous frame (red points in column (b)), but invisible in the current frame (column (a)). The right arm has invalid *M*_1_ (column (d)). The re-initialization result (re-do GLTP with a template where the right arm is set as invisible) is shown in column (e). (2) In the third row (Case II), the left arm is trapped in the torso, and the right arm has an enlarged volume to cover the points from both arms (column (c)). The left arm has invalid *M*_1_, and the right arm has invalid *M*_2_ (column (d)). Column (e) shows the re-initialization result with the recovered left arm after GLTP registration using the “T-pose” template. (3) In the fourth row (Case III), both left and right arms and part of the torso are missing, caused by large self-occlusions. Correspondence estimation results are invalid (column (c)), leading to invalid *M*_1_ and *M*_2_ for most segments (column (d)). Column (e) shows the pose estimation result by using pose continuity and physical constraints.

#### 4.2.3. Experimental Results: Pose Estimation

We evaluate our proposed pose estimation framework in two settings. The first one treats each frame independently with the same “T-pose” template (the same as [49]), and the other one involves the tracking strategy by updating the template sequentially via visible point extraction from the previous pose estimation result. Out of 28 depth sequences, the subject keeps a stable view point in all but two (24 and 27) sequences. In Sequences 24 and 27, the subject undergoes significant view changes. In the the first setting, the frontal view “T-pose” template is used at each frame when all body segments are visible, and the large pose variation between the template and target models may lead to inaccurate pose estimation results in some challenging frames. The pose tracking scheme introduced in the second setting is expected to be more effective and accurate to deal with sequential depth data where segment volume validation plays an important rule to ensure a smooth and valid tracking result. Some qualitative results on four selected sequences are shown in Figure 21, where the first and second rows show the results from two pose estimation settings (without and with tracking).

**Figure 20:**
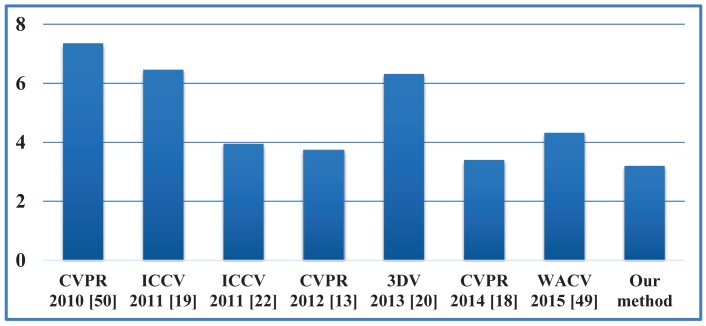
Comparative analysis of the joint estimation error (cm).

**Figure 21:**
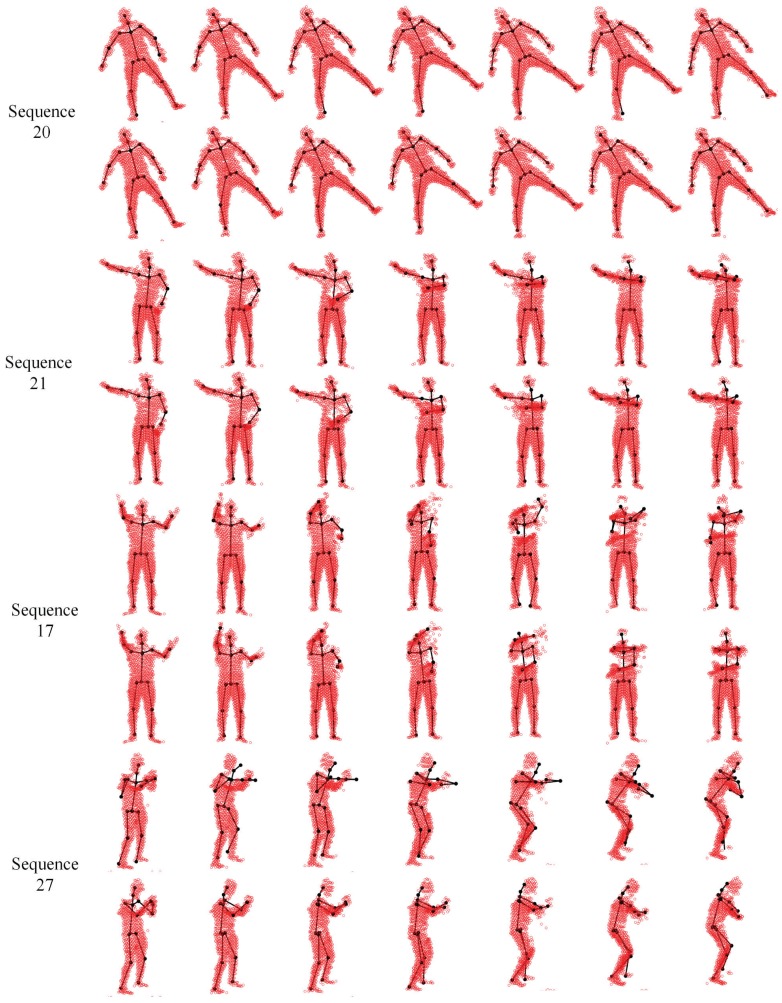
Pose estimation results for four SMMC-10 sequences. For each sequence, the first and second rows show the results without tracking and those with tracking, respectively.

Our proposed framework is also compared against some recent state-of-the-art algorithms [13,18–20,22,49,50] in terms of the error between each estimated joint and its corresponding ground-truth marker. Given a sequence with *N_f_* frames and *N_j_* joints, the joint estimation error is defined as:

(13) $$e=\frac{1}{N_{f}N_{j}}\overset{N_{f}}{\underset{k=1}{\text{∑}}}\overset{N_{j}}{\underset{i=1}{\text{∑}}}\left\|J_{i}^{k}-M_{i}^{k}-O_{i}\right\|$$

where $J_{i}^{k}$ and $M_{i}^{k}$ are the estimated position and the ground-truth marker position of the *i*-th joint in the *k*-th frame. Due to the inconsistency between the definition of joints between the template skeleton and the configuration of markers, we need to remove a constant offset *O_i_* at each joint that is computed along the local segment based on 20 manually-selected frames. Figure 18c,d show the initial pose from the depth image and the learned subject-specific shape model with labeled segments and the estimated skeleton, respectively. The quantitative comparison against several recent algorithms in terms of the position error (averaged over all frames from 28 sequences) is shown in Figure 20. The accuracy of pose estimation is significantly improved compared with that in [49] (4.3 cm) due to the tracking capability, including visible point extraction and segment volume validation. The average joint position error is 3.2 cm, which outperforms all existing methods, including the most recent work [18] (3.4 cm).

### 4.3. Computational Complexity

The computational loads of the three registration algorithms (CPD, GLTP and SAICP) involved in the proposed framework are shown in Table 1. CPD is only performed once for personalized body shape initialization, and GLTP shares a similar EM algorithm as CPD. Their computational costs could be reduced by using fast implementations [34], then the cost for computing P in CPD and GLTP, which involves the calculation of the sum of exponentials, could be reduced from *O*(*MN*) to *O*(*M* + *N*). Pose tracking reduces the computational load of GLTP significantly By using the low-rank matrix approximation, the cost for solving the linear system defined in Equation (7) to find **W** in GLTP could be lowered from *O*(*M*^3^) to *O*(*R*^3^), where *R* ≪ *M* is the lower rank value. Using the K-D tree to search for nearest neighbors [24] in GLTP and SAICP, the cost could be further decreased form *O*(*M*^2^) to *O*(*M* log(*M*)).

In practice, the algorithm speed depends on the numbers of points in the template and targets (around 1000 points for each) as well as the iteration numbers in GLTP and SAICP. The tracking strategy greatly reduces the number of iterations needed in GLTP by providing a good initialization for sequential registration. Also, due to reliable correspondence estimation from GLTP, SAICP only needs a few iterations to converge for each segment. Our algorithm was implemented in an un-optimized MATLAB code. For depth sequences, the running time is around 10 s (without tracking) or 3 s (with tracking) per frame on a PC with Intel i7 CPU 3.40 GHz and 32GB RAM. The proposed algorithm can be speeded up significantly by GPU acceleration with C/C++ implementation.

## 5. Conclusions

We propose a new generative framework for 3D human pose estimation from point sets captured by laser scanners or depth cameras. Without any initialization or training data, the proposed approach can handle complex articulated motions by combining two registration techniques in a complimentary way. One is the global-local topology preservation (GLTP) algorithm, which aims at non-rigid and articulated deformation, and the other one is the segment-aware AICP (SAICP) algorithm that takes advantage of reliable correspondence estimation by GLTP for articulate pose estimation. Furthermore, to handle sequential depth data, which may have missing data caused by self-occlusions and view changes, we introduce an efficient tracking strategy where two new techniques, e.g., visible point extraction and segment volume validation, are developed to support sequential registration. The experimental results on benchmark 3D laser scan and depth datasets demonstrate the effectiveness of the proposed framework.

## Figures and Tables

**Table 1:** Computational complexity of three registration algorithms.

**Algorithms**	**Computational Complexity**
CPD	*O*(*MN*) + *O*(*M*^3^)
GLTP (without tracking)	*O*(*MN*) + *O*(*M*^3^)
GLTP (with tracking)	*O*(*MN*) + *O*(*M*^3^) + *O*(*M*^2^) + *O*(*MK*^3^)
SAICP	$O\left(M_{\psi }^{2}\right)$

*M* and *N* are the number of points in the template and target, respectively; *K* is the number of local linear embedding (LLE) neighbors in GLTP; *M*_ψ_ is the number of points in a selected rigid part ψ in SAICP.
